# Restorative free time and psychological distress among employed family caregivers of individuals with cognitive impairment: a cross-sectional study

**DOI:** 10.3389/fpsyg.2026.1898843

**Published:** 2026-10-26

**Authors:** Tomomi Matsumoto, Naoaki Ohkubo, Hisashi Eguchi, Yoshihisa Fujino, Reiji Yoshimura, Atsuko Ikenouchi

**Affiliations:** 1 Department of Psychiatry, School of Medicine, University of Occupational and Environmental Health, Kitakyushu, Japan; 2 Department of Environmental Epidemiology, Institute of Industrial Ecological Sciences, University of Occupational and Environmental Health, Kitakyushu, Japan; 3 Department of Mental Health, Institute of Industrial Ecological Sciences, University of Occupational and Environmental Health, Kitakyushu, Japan; 4 Medical Center for Dementia, Hospital of the University of Occupational and Environmental Health, Kitakyushu, Japan

**Keywords:** caregivers, cognitive impairment, dementia, psychological distress, restorative free time, work-life balance

## Abstract

**Background:**

Employed family caregivers of individuals with cognitive impairment often need to balance work responsibilities, caregiving tasks, and their own daily lives. Although caregiving burden is commonly discussed in terms of time spent caregiving, less attention has been paid to whether caregivers have enough time to recover from these demands.

**Objective:**

This study examined the associations of daily time-use factors, including restorative free time, sleep duration, working hours, and caregiving duration, with psychological distress among employed family caregivers of individuals with cognitive impairment.

**Methods:**

A cross-sectional study was conducted as part of the Sustaining for Aging Dementia Relatives Support and Employment project in Japan. Questionnaires were distributed to family caregivers, and data from 210 employed caregivers were analyzed. Psychological distress was assessed using the Kessler 6 scale. Modified Poisson regression with robust variance estimation was used to estimate prevalence ratios (PRs) and 95% confidence intervals (CIs) for the associations between daily time use and psychological distress. In supplementary analyses, logistic regression was used to explore factors associated with severely limited restorative free time (<1 h/day), including care recipient characteristics such as functional status and long-term care certification.

**Results:**

Caregivers with less than 1 h of restorative free time per day had a significantly higher prevalence of psychological distress than those with 3 h or more. Short sleep duration (<6 h/day) was also associated with a higher prevalence of psychological distress, whereas working hours and caregiving duration were not significantly associated with psychological distress after multivariable adjustment.

**Conclusion:**

Severely limited restorative free time and short sleep duration were associated with a higher prevalence of psychological distress among employed family caregivers. Limited restorative free time may therefore serve as a potential marker of psychological distress in this population. However, the cross-sectional design does not establish the temporal or causal direction of these associations.

## Introduction

Population aging is progressing rapidly worldwide. In Japan, declining birth rates and increased life expectancy have contributed to a growing number of older adults who require medical and long-term care. As a result, family members are increasingly taking on caregiving roles, and informal caregiving has become an essential part of long-term care systems (World Health Organization, 2017; OECD, 2021).

Providing care for a family member can place considerable physical, emotional, and social demands on caregivers (Pinquart and Sörensen, 2007). Family caregivers often assist with daily activities, supervise care recipients, and respond to behavioral and psychological symptoms, especially among individuals with dementia or other cognitive impairments (Baumgarten, 1989; Schulz and Sherwood, 2008). In this study, cognitive impairment refers to dementia and mild cognitive impairment (MCI). Caring for individuals with dementia or MCI may place sustained demands on family caregivers, depending on the severity of cognitive and functional impairment (Schulz and Martire, 2004; Schulz et al., 2020). Previous studies have shown that caregivers tend to experience greater psychological distress than non-caregivers (Pinquart and Sörensen, 2003; Vitaliano et al., 2003).

These difficulties may become even more pronounced when caregivers are also employed. Employed family caregivers must simultaneously manage paid work, caregiving responsibilities, and other daily obligations, which can create role conflict and time pressure and may increase the risk of psychological strain or work-related difficulties (Earle and Heymann, 2011; Yamada and Shimizutani, 2015). They must also allocate finite daily time across work, caregiving, sleep, and personal recovery. The coexistence of work and caregiving demands may therefore constrain opportunities for psychological and physical recovery. Although caregiver research has often focused on caregiving demands and burden, less is known about the role of recovery-related discretionary time in psychological distress among family caregivers who are simultaneously engaged in paid employment. Examining restorative free time alongside other major domains of daily time use may therefore provide a more comprehensive understanding of psychological distress in employed family caregivers.

Research on caregiver burden has traditionally focused on the amount of time spent providing care. However, caregiving hours alone may not fully reflect caregivers’experiences. Family caregiving may involve contingent and unpredictable demands that constrain participation in activities outside caregiving, even when some time appears to be available (Altomonte, 2016). Even among individuals with similar caregiving time, perceived burden and psychological distress can differ depending on personal and environmental circumstances (Pearlin et al., 1990; Schulz and Sherwood, 2008). This suggests that caregiver well-being may depend not only on the amount of time spent caregiving but also on whether caregivers have opportunities to recover from ongoing demands within their daily lives.

One factor that may be particularly important is restorative free time. In this study, restorative free time refers to the perceived amount of discretionary time available for psychological or physical recovery from daily demands. This concept is related to research on recovery experiences, including psychological detachment and relaxation (Sonnentag and Fritz, 2007; Sonnentag, 2018), as well as to restorative processes discussed in environmental psychology (Kaplan, 1995; Hartig et al., 2003). However, restorative free time is conceptually distinct from these recovery experiences. Whereas psychological detachment and relaxation refer to experiences that may occur during non-work time, restorative free time in the present study focuses on the perceived availability of time that can be used for recovery. It is also more specific than discretionary time in general because it explicitly refers to time available for one’s mental or physical well-being. Thus, restorative free time in this study represents a perceived temporal opportunity for recovery rather than the quality or content of the recovery experience itself.

To date, relatively few studies have examined multiple aspects of daily time use together, such as caregiving time, work hours, sleep duration, and restorative free time, in relation to psychological distress among employed caregivers. Therefore, this study examined the associations between these time-use factors and psychological distress among employed family caregivers of individuals with cognitive impairment. We expected that shorter restorative free time and shorter sleep duration would be associated with greater psychological distress. We also examined working hours and caregiving duration as other major components of daily time use in relation to psychological distress.

## Materials and methods

### Study design and participants

This cross-sectional study was conducted as part of the Sustaining for Aging Dementia Relatives Support and Employment (SASAE) project (Ikenouchi et al., 2025). The study population consisted of family caregivers of patients diagnosed with dementia or MCI who attended the outpatient clinic for dementia at the hospital of the University of Occupational and Environmental Health, Japan.

Eligible patients and their family caregivers were approached for study participation on an ongoing basis throughout the recruitment period from October 2021 to December 2025. During the recruitment period, 360 patient–family caregiver pairs were approached for participation and received an explanation of the study. Among them, 346 caregivers provided written informed consent and were subsequently given a questionnaire. Questionnaires were returned by 261 caregivers. One caregiver was excluded because both daily working hours and the number of working days per week were reported as zero, indicating that the participant was not employed.

Of the remaining 260 employed caregivers, 50 were excluded because of missing data for one or more analysis variables. Missing responses occurred most frequently for daily caregiving duration (*n* = 22), whereas missing responses for the remaining variables were infrequent (generally one to five participants per variable), with no apparent systematic pattern of missingness. Consequently, 210 employed caregivers with complete data were included in the final analysis. To assess potential selection bias related to the complete-case analysis, the available baseline characteristics of the 210 included and 50 excluded employed caregivers were compared descriptively. Further information on the care recipients was collected from medical records, including age, sex, diagnosis, level of long-term care certification, and results of cognitive and functional assessments, such as the Mini-Mental State Examination (MMSE) and the Physical Self-Maintenance Scale (PSMS). Clinical information on care recipients was generally extracted from medical records at the time the caregiver provided informed consent. The investigators who extracted the clinical information were not blinded to the caregivers’ questionnaire responses.

This study was approved by the Ethics Committee of the University of Occupational and Environmental Health, Japan (No. R3–010), and written informed consent was obtained from all participants after they received a full explanation of the study.

### Assessments

Data from caregivers were collected using a self-administered questionnaire, which included items on age, sex, marital status, educational level, job type, employment conditions, cohabitation status, sleep duration, working hours, caregiving hours, and lifestyle habits (e.g., smoking and alcohol consumption). Information on care recipients was obtained from medical records and included age, sex, diagnosis, and MMSE and PSMS scores. Cognitive function was assessed using the MMSE, a widely used measure of global cognitive function, with lower scores indicating greater cognitive impairment. Basic activities of daily living were assessed using the PSMS (Lawton and Brody, 1969), with higher scores indicating better functional status (i.e., greater independence).

### Psychological distress

Psychological distress was assessed using the Japanese version of the K6, a brief and widely used screening instrument for non-specific psychological distress (Kessler et al., 2002). The validity and screening performance of the Japanese version have been well established (Furukawa et al., 2008; Sakurai et al., 2011). The K6 comprises six items assessing the frequency of psychological symptoms during the past 30 days, including nervousness, hopelessness, restlessness, effortfulness, depressive mood, and worthlessness. Each item is rated on a five-point Likert scale, ranging from 0 (none of the time) to 4 (all of the time), with total scores ranging from 0 to 24. A cutoff score of ≥5 has been proposed for screening mood and anxiety disorders in the Japanese general population (Sakurai et al., 2011), whereas a higher cutoff score of ≥13 has been used to screen for serious mental illness (Kessler et al., 2003). In the SASAE project, K6 scores of ≥5 and ≥13 were used as thresholds for mild and severe psychological distress, respectively, and 44% and 7% of caregivers met these thresholds at baseline (Ikenouchi et al., 2025). Given the small proportion of caregivers meeting the ≥13 threshold, a cutoff score of ≥5 was used to define psychological distress in the present study.

### Time-use variables

Daily time use was assessed using self-reported measures of average daily working hours, caregiving hours, sleep duration, and restorative free time. Working hours, caregiving hours, and sleep duration were self-reported as average daily amounts.

Restorative free time was assessed using a self-reported single-item question developed for this study based on the conceptual definition of discretionary time for recovery. This measure was conceptually grounded in prior research on recovery and discretionary time (Sonnentag and Fritz, 2007, 2015), although it has not been previously validated as a standardized scale. The item was not pilot tested prior to this study. Instead, it was developed through multidisciplinary discussions among psychiatrists, clinical psychologists, epidemiologists, and dementia specialists within the SASAE project team. No formal scale-development procedure or psychometric validation was conducted, and formal content validity was not established. Participants were asked, “On average, how much time per day do you have that you can use at your own discretion for your mental or physical well-being?”

Participants reported the average number of hours per day. For the purposes of analysis, restorative free time was categorized into three groups: <1 h/day, 1– < 3 h/day, and ≥3 h/day. The daily time-use variables were collected as continuous measures and categorized before analysis based on the study design and the observed distribution of the data to facilitate interpretation of the results. These cutoffs were not based on established clinical or validated thresholds. This variable was intended to capture perceived opportunities for recovery-related discretionary time, rather than the specific content or quality of those activities. Because all time-use variables were self-reported, subjective interpretation may have influenced the measurements.

### Statistical analysis

Associations between restorative free time, sleep duration, working hours, and caregiving hours and psychological distress were examined using modified Poisson regression with robust variance estimation to estimate prevalence ratios (PRs) and 95% confidence intervals (CIs), given the relatively high prevalence of psychological distress. A complete-case analysis was performed, and no imputation of missing data was conducted.

In the primary analysis, restorative free time, sleep duration, working hours, and caregiving duration were entered simultaneously into the regression models. For each time-use variable, the first model was adjusted for caregiver age and sex and the other three time-use variables. The fully adjusted model additionally included care recipient age, sex, diagnosis, MMSE and PSMS scores, and level of long-term care certification, as well as caregiver marital status, educational level, job type, number of working days, presence of night or shift work, cohabitation status with the care recipient, smoking, and alcohol consumption. These covariates were selected *a priori* based on previous literature and theoretical relevance. The fully adjusted model included 34 parameters, with 176 residual degrees of freedom. Spearman rank correlations among the four daily time-use variables are presented in Supplementary Table 3. Variance inflation factors (VIFs) for the fully adjusted model ranged from 1.21 to 2.86 (mean VIF, 1.71). Because daily time is finite and the four time-use variables may be interrelated, sensitivity analyses were conducted in which each time-use variable was examined separately without mutual adjustment for the other time-use variables. The first model was adjusted for caregiver age and sex. A parsimonious model additionally included care recipient diagnosis, MMSE score, level of long-term care certification, and caregiver cohabitation status; these additional covariates were selected based on their presumed relevance to psychological distress rather than according to a strictly prespecified selection procedure. To address the potential subjectivity of this covariate selection, additional models included a fully adjusted model, a patient-factor model, and a caregiver-factor model, without mutual adjustment for the other time-use variables. As a sensitivity analysis, restorative free time was also examined as a continuous variable. This analysis was conducted using a model adjusted for caregiver age and sex and a multivariable model adjusted for the covariates included in the fully adjusted model, without adjustment for the other time-use variables. In addition, exploratory univariable logistic regression analyses were performed to explore factors associated with having less than 1 h per day of restorative free time. These analyses were exploratory and unadjusted and were not intended to identify independent predictors of limited restorative free time. All statistical analyses were performed using Stata Statistical Software, Release 18 (StataCorp LLC). A two-sided *p*-value < 0.05 was considered statistically significant.

## Results

Caregivers’characteristics are presented in Table 1. Among the participants, 68 were men and 142 were women, with a mean age of 54.3 years. Regarding marital status, 76% were married, and 40% lived with the care recipient. Regarding educational attainment, 29% had completed university or graduate school, while 36% had graduated from vocational schools or junior colleges.

**TABLE 1:** Characteristics of caregivers according to restorative free time.

Characteristic	Total	≥ 3 h	1–<3 h	< 1 h
	(N = 210)	(n = 58)	(n = 135)	(n = 17)
Age, mean (SD)	54.3 (8.5)	56.6 (9.6)	53.1 (7.8)	55.4 (8.3)
Sex, male, n (%)	68 (32%)	18 (31%)	47 (35%)	3 (18%)
Currently married, n (%)	159 (76%)	46 (79%)	103 (76%)	10 (59%)
Educational attainment				
Junior high school/high school	73 (35%)	21 (36%)	49 (36%)	3 (18%)
Vocational school/junior college/technical college	76 (36%)	20 (34%)	48 (36%)	8 (47%)
University/graduate school	61 (29%)	17 (29%)	38 (28%)	6 (35%)
Job type, n (%)				
Mainly desk work (clerical or computer work)	87 (41%)	16 (28%)	66 (49%)	5 (29%)
Mainly talking to people (customer service, sales, selling, etc.)	48 (23%)	17 (29%)	29 (21%)	2 (12%)
Mainly manual labor (production-site work, physical work, nursing care, etc.)	68 (32%)	23 (40%)	37 (27%)	8 (47%)
Other	7 (3%)	2 (3%)	3 (2%)	2 (12%)
Working days, mean (SD)	4.9 (0.9)	4.8 (1.1)	4.9 (0.9)	4.9 (0.8)
Night/rotating shifts, n (%)	17 (8%)	5 (9%)	9 (7%)	3 (18%)
Psychological distress (K6 ≥ 5), n (%)	85 (40%)	21 (36%)	52 (39%)	12 (71%)
Living with care recipient, n (%)	84 (40%)	25 (43%)	54 (40%)	5 (29%)
Sleep duration, n (%)				
< 6 h	53 (25%)	9 (16%)	35 (26%)	9 (53%)
6–<8 h	135 (64%)	38 (66%)	89 (66%)	8 (47%)
≥ 8 h	22 (10%)	11 (19%)	11 (8%)	0 (0%)
Working hours, n (%)				
≤ 6 h	59 (28%)	25 (43%)	31 (23%)	3 (18%)
> 6–≤8 h	114 (54%)	25 (43%)	78 (58%)	11 (65%)
> 8 h	37 (18%)	8 (14%)	26 (19%)	3 (18%)
Caregiving duration, n (%)				
≤ 1.5 h	52 (25%)	11 (19%)	37 (27%)	4 (24%)
> 1.5–≤3 h	53 (25%)	11 (19%)	35 (26%)	7 (41%)
> 3–≤8 h	57 (27%)	17 (29%)	38 (28%)	2 (12%)
> 8 h	48 (23%)	19 (33%)	25 (19%)	4 (24%)
Current smoker, n (%)	28 (13%)	7 (12%)	21 (16%)	0 (0%)
Alcohol consumption, n (%)				
None	81 (39%)	22 (38%)	49 (36%)	10 (59%)
≥ 1/month	79 (38%)	17 (29%)	58 (43%)	4 (24%)
Daily	50 (24%)	19 (33%)	28 (21%)	3 (18%)

Other includes participants who reported more than one job type (desk work and interpersonal work, *n* = 2; desk work and manual labor, *n* = 1; interpersonal work and manual labor, *n* = 4). There were no missing values for the variables presented in this table among the 210 participants included in the complete-case analysis. K6, Kessler 6-item Psychological Distress Scale

Job types were distributed as follows: mainly desk work (clerical or computer work; 41%), mainly talking to people (customer service, sales, selling, etc.; 23%), and mainly labor (work at production sites, physical work, nursing care, etc.; 32%). Concerning working hours, 54% worked more than 6 h and up to 8 h per day, which was the most common category, while 28% worked 6 h or less. Most caregivers (92%) worked only day shifts.

Regarding lifestyle factors, 13% of participants were current smokers. Regarding alcohol consumption, 38% reported drinking alcohol at least once per month, and 24% reported drinking alcohol daily. Sleep duration was most commonly reported as 6– < 8 h per night (64%). Caregiving time was relatively evenly distributed across categories, with approximately 25% of participants in each group: ≤1.5 h, >1.5– ≤ 3 h, >3– ≤ 8 h, and >8 h per day.

The characteristics of the care recipients are presented in Table 2. The mean age of the care recipients was 79.9 years, and 68% were women. Regarding diagnoses, 34% had Alzheimer’s disease, 37% had mild cognitive impairment, and 29% had other dementia-related disorders.

**TABLE 2:** Characteristics of care recipients according to caregivers’restorative free time.

		Restorative free time		
Variable	Total	≥3 h/day	1– < 3 h/day	<1 h/day
	(*N* = 210)	(*n* = 58)	(*n* = 135)	(*n* = 17)
Age, years (mean ± SD)	79.9 (7.3)	80.4 (6.7)	79.5 (7.6)	80.9 (6.3)
Men, *n* (%)	67 (32%)	22 (38%)	42 (31%)	3 (18%)
Diagnosis, n (%)				
Alzheimer’s dementia	71 (34%)	16 (28%)	48 (36%)	7 (41%)
Mild cognitive impairment	78 (37%)	22 (38%)	51 (38%)	5 (29%)
Other	61 (29%)	20 (34%)	36 (27%)	5 (29%)
Long-term care certification, n (%)				
None	93 (44%)	25 (43%)	64 (47%)	4 (24%)
Support level 1–2	40 (19%)	14 (24%)	24 (18%)	2 (12%)
Care level 1–5	77 (37%)	19 (33%)	47 (35%)	11 (65%)
MMSE (mean ± SD)	21.5 (5.5)	21.7 (5.6)	21.6 (5.5)	20.5 (5.6)
PSMS (mean ± SD)	4.5 (1.8)	4.8 (1.5)	4.5 (1.8)	3.2 (2.1)

Higher PSMS scores indicate better functional status (greater independence in basic activities of daily living). MMSE, Mini-Mental State Examination; PSMS, Physical Self-Maintenance Scale. There were no missing values for the variables presented in this table among the 210 participants included in the complete-case analysis.

Regarding the use of long-term care insurance services, 44% were not using long-term care services, 19% were certified as requiring support, and 37% were certified as requiring long-term care. The mean (SD) MMSE score was 21.5 (5.5), and the mean (SD) PSMS score was 4.5 (1.8).

Available characteristics of the 210 caregivers included in the complete-case analysis and the 50 excluded caregivers are shown in Supplementary Tables 4, 5. Although many characteristics were similar between the groups, excluded caregivers were older and less likely to have desk-based employment than included caregivers.

The associations between daily time use and psychological distress are presented in Table 3. PRs were estimated using modified Poisson regression analysis. The prevalence of psychological distress (K6 ≥ 5) was 36.2% (21/58) among caregivers with ≥3 h/day of restorative free time, 38.5% (52/135) among those with 1– < 3 h/day, and 70.6% (12/17) among those with <1 h/day. Compared with caregivers with ≥3 h/day, those with <1 h/day had a higher prevalence of psychological distress in the time-use-, age-, and sex-adjusted analysis (PR = 1.82, 95% CI: 1.04–3.19, *p* = 0.036) and in the fully adjusted analysis (adjusted PR = 1.93, 95% CI: 1.07–3.49, *p* = 0.030). Compared with caregivers with ≥3 h/day of restorative free time, those with <1 h/day had a higher prevalence of psychological distress in the time-use-, age-, and sex-adjusted analysis (PR = 1.93, 95% CI: 1.13—3.31, *p* = 0.016) and in the fully adjusted analysis (adjusted PR = 1.95, 95% CI: 1.07–3.54, *p* = 0.028). Caregivers with 1–<3 h/day did not show a significant difference compared with those with ≥3 h/day. Caregivers who slept <6 h/day had a significantly higher prevalence of psychological distress compared with those who slept 6–<8 h/day (PR = 1.64, 95% CI: 1.17–2.30, *p* = 0.004). This association remained significant in the fully adjusted analysis (adjusted PR = 1.78, 95% CI: 1.24–2.56, *p* = 0.002). Working hours and caregiving duration were not significantly associated with psychological distress after multivariable adjustment.

**TABLE 3:** Association between daily time use and psychological distress.

Variable	Time-use-, age-, and sex-adjusted model^1^			Multivariable model^2^		
	PR	95% CI	*p* value	PR	95% CI	*p* value
Restorative free time						
≥3 h/day	Reference			Reference		
1–<3 h/day	1.14	0.77–1.73	0.503	1.06	0.69–1.64	0.777
<1 h/day	1.93	1.13–3.31	0.016	1.95	1.07–3.54	0.028
Sleep duration						
<6 h/day	1.64	1.17–2.30	0.004	1.78	1.24–2.56	0.002
6–<8 h/day	Reference			Reference		
≥8 h/day	1.19	0.68–2.09	0.546	1.17	0.67–2.06	0.583
Working hours						
≤6 h/day	Reference			Reference		
>6–≤8 h/day	1.00	0.68–1.47	0.988	1.08	0.72–1.63	0.713
>8 h/day	1.16	0.69–1.94	0.584	1.16	0.65–2.11	0.603
Caregiving duration						
≤1.5 h/day	Reference			Reference		
>1.5–≤3 h/day	0.90	0.52–1.53	0.690	0.85	0.51–1.43	0.540
>3–≤8 h/day	1.06	0.64–1.76	0.824	0.95	0.53–1.68	0.848
>8 h/day	1.55	0.95–2.53	0.078	1.45	0.75–2.79	0.272

^1^Adjusted for age, sex, and the other three daily time-use variables.

^2^Additionally adjusted for care recipient age and sex, diagnosis, MMSE, PSMS, long-term care insurance status, cohabitation, marital status, education, job type, number of working days, shift work, smoking, and alcohol consumption.

Although caregivers who provided ≥8 h/day of caregiving showed an increased prevalence of psychological distress in the analysis adjusted for age, sex, and the other three time-use variables, this association was not significant in the multivariable model. Sensitivity analyses, in which each time-use variable was examined separately without mutual adjustment for the other time-use variables, showed generally similar patterns across the five models (Supplementary Table 1). For restorative free time <1 h/day, PRs ranged from 1.61 to 2.48 across the models, with 95% CIs including 1.00 only in the patient-factor model (PR = 1.61, 95% CI: 0.95–2.74). Short sleep duration (<6 h/day) was consistently associated with a higher prevalence of psychological distress across all five models, with PRs ranging from 1.63 to 1.96.

In the sensitivity analysis treating restorative free time as a continuous variable, each additional hour of restorative free time was associated with a PR of 0.88 for psychological distress in the age- and sex-adjusted model (95% CI: 0.77–1.00, *p* = 0.050). In the multivariable model without adjustment for the other time-use variables, the corresponding PR was 0.88 (95% CI: 0.77–1.01, *p* = 0.078). Supplementary Table 2 presents the results of the univariable logistic regression analyses examining factors associated with severely limited restorative free time (<1 h/day).

Among caregiver characteristics, psychological distress measured by the K6 score was significantly associated with limited restorative free time (odds ratio [OR] = 1.12, 95% CI: 1.03–1.23, *p* = 0.010). No significant associations were observed for age, sex, marital status, educational background, job type, number of working days, or shift work.

Regarding care recipient characteristics, higher levels of long-term care needs (care level 1–5) were significantly associated with having <1 h/day of restorative free time (OR = 3.53, 95% CI: 1.25–9.96, *p* = 0.017). In addition, lower PSMS scores, reflecting poorer functional status, were significantly associated with reduced restorative free time (OR = 0.71, 95% CI: 0.56–0.90, *p* = 0.004). No significant associations were found for care recipient age, sex, diagnosis, or cognitive function as measured by the MMSE.

These findings represent unadjusted associations and should not be interpreted as independent predictors. Moreover, because only 17 caregivers were in the <1-h/day group, the precision of estimates from these exploratory analyses is limited, and the findings should be interpreted cautiously.

## Discussion

This study examined how different aspects of daily time use were related to psychological distress among employed family caregivers of individuals with cognitive impairments. Among the time-use variables examined, restorative free time and short sleep duration were significantly associated with psychological distress, whereas caregiving hours and work hours were not significantly associated after adjustment. In particular, caregivers with less than 1 h of restorative free time per day were more likely to experience psychological distress than those with 3 h or more. These findings suggest that opportunities for recovery in daily life may be especially important for caregiver mental health.

The present findings are generally consistent with previous studies reporting elevated psychological distress among caregivers (Pinquart and Sörensen, 2003; Schulz and Sherwood, 2008). At the same time, this study extends prior research by focusing on the potential role of recovery opportunities in everyday life. Earlier studies have often emphasized caregiving intensity, such as hours spent providing care, as a major contributor to caregiver burden (Adelman et al., 2014). In the present study, restorative free time of <1 h/day was associated with a higher prevalence of psychological distress, whereas caregiving duration was not significantly associated with psychological distress after multivariable adjustment. This interpretation is also consistent with previous evidence showing that caregiving hours alone do not fully explain differences in caregiver burden or mental health outcomes (Pinquart and Sörensen, 2003).

The observed association between limited restorative free time and psychological distress can be interpreted in light of several theoretical frameworks, although the cross-sectional design does not allow us to determine the mechanisms underlying this association. The Effort–Recovery Model proposes that insufficient recovery contributes to the accumulation of strain over time (Meijman and Mulder, 1998). This interpretation is also consistent with the Conservation of Resources Theory, which proposes that psychological stress may arise when valued resources are threatened, lost, or insufficiently replenished (Hobfoll, 1989). Similarly, environmental psychology has emphasized the importance of restorative experiences for replenishing cognitive and emotional resources (Kaplan, 1995; Hartig et al., 2003). Previous empirical studies have shown that experiences such as psychological detachment, relaxation, and a sense of control are associated with better well-being (Sonnentag and Fritz, 2007, 2015). This perspective is also consistent with the concept of allostatic load, which refers to the cumulative physiological burden associated with repeated or prolonged activation of stress-response systems (McEwen, 1998). In employed family caregivers, limited opportunities for recovery may contribute to the accumulation of strain associated with balancing work and caregiving responsibilities. These frameworks provide possible interpretations of the observed association but were not directly tested in the present study.

The present study suggests that these perspectives may also be relevant for employed caregivers who must continuously manage competing demands across work, caregiving, and daily life.

One possible explanation is that restorative free time reflects whether caregivers are able to step away, even briefly, from ongoing caregiving and work-related demands. Caregiving responsibilities are often continuous, leaving some caregivers with few opportunities to mentally disengage during daily life. When opportunities for recovery are limited, psychological strain may gradually accumulate over time. Therefore, severely limited restorative free time may reflect limited opportunities for recovery from ongoing demands and may serve as a marker of caregivers at increased risk of psychological distress.

The supplementary analyses further showed associations between the functional status and care needs of care recipients and caregivers’restorative free time. Higher PSMS scores, indicating better functional status (greater independence in basic activities of daily living), were associated with lower odds of having severely limited restorative free time. Conversely, lower PSMS scores, reflecting greater functional dependency, were associated with a greater likelihood of having severely limited restorative free time. This finding is consistent with previous research suggesting that care recipient characteristics, including functional limitations and care needs, contribute to caregiver burden (Etters et al., 2008). These findings indicate that restorative free time was associated with caregiving intensity and care recipient dependency; however, because these factors and restorative free time were assessed concurrently, the temporal or causal direction of these associations cannot be determined. Additional analyses showed that short sleep duration was associated with psychological distress, which is consistent with prior research linking insufficient sleep to poorer mental health outcomes (Baglioni et al., 2016). In contrast, caregiving hours and work hours were not independently associated with psychological distress after adjustment. This does not necessarily mean that these factors are unimportant. In this context, restorative free time may serve as a potential marker of psychological distress among employed family caregivers.

From a practical perspective, the observed association between severely limited restorative free time and psychological distress suggests that opportunities for recovery may warrant consideration in future research on caregiver support. Potential approaches, such as respite care, flexible work arrangements, and support for balancing work and caregiving responsibilities, warrant evaluation in future intervention studies to determine whether they can increase opportunities for restorative free time and improve caregiver well-being.

Future research should further examine the qualitative aspects of restorative free time, including how caregivers spend this time and which activities are most effective for recovery. Longitudinal and intervention studies will also be important for clarifying causal relationships and identifying effective approaches for supporting caregiver well-being.

This study has several limitations. First, because of the cross-sectional design, causal relationships between restorative free time and psychological distress cannot be determined. Reverse causation is also possible, as caregivers experiencing greater psychological distress may perceive or report having less restorative free time (Lazarus and Folkman, 1984; Pearlin et al., 1990). Longitudinal studies are needed to clarify the temporal relationship between restorative free time and psychological distress.

Second, all time-use variables, including restorative free time, work hours, caregiving hours, and sleep duration, were assessed using self-reported measures and may therefore be affected by recall bias or subjective interpretation. In addition, restorative free time was measured using a single-item question rather than a previously validated standardized scale, which may limit measurement precision. These measurement limitations may have resulted in measurement error or misclassification of the time-use exposures, although the direction and magnitude of any resulting bias are uncertain. The measurement properties of the restorative free time item, including content validity, have not been formally established. In addition, the cutoffs used to categorize restorative free time were not based on established clinical or validated thresholds.

Third, several potentially important factors, including behavioral and psychological symptoms of dementia, subjective caregiver burden, and social support, were not explicitly included in the analytical model. Some of these variables may lie on the causal pathway linking care recipients’functional dependency with caregivers’available time and psychological outcomes. Therefore, their inclusion requires careful conceptual consideration. Similarly, although economic information such as household income was collected, it was not included in the final analytical model for conceptual reasons.

Fourth, this study was conducted at a single university hospital outpatient dementia clinic in Japan, which may limit the generalizability of the findings to other settings and populations. Therefore, the findings may not be generalizable to unemployed caregivers, caregivers in rural settings, or those without access to specialist dementia services. In addition, only employed family caregivers with complete questionnaire data were included in the analyses. Furthermore, caregivers experiencing greater caregiving burden may have been less likely to participate or to return complete questionnaires. Notably, the subgroup with <1 h/day of restorative free time included only 17 participants. The small number of participants in this category may have reduced the precision and stability of the corresponding estimates. Accordingly, the findings for this subgroup should be interpreted with caution. In addition, given the modest sample size relative to the number of covariates included in the fully adjusted model, the possibility of overfitting and unstable estimates cannot be excluded.

Fifth, because daily time is finite, restorative free time, sleep duration, working hours, and caregiving duration are inherently interrelated. However, sensitivity analyses in which each time-use variable was examined separately without mutual adjustment for the other time-use variables yielded results that were generally consistent with those of the primary multivariable analysis. Therefore, the observed associations should be interpreted in light of this interdependence.

Finally, participants were recruited over a 4-year period (October 2021 to December 2025). During this period, changes in external circumstances, including the later stages of the COVID-19 pandemic and subsequent changes in caregiving and work environments, may have influenced caregivers’experiences and time use. Therefore, these factors should be considered when interpreting the findings.

## Conclusion

In this cross-sectional study of employed family caregivers of individuals with cognitive impairment, very limited restorative free time (<1 h/day) and short sleep duration (<6 h/day) were associated with a higher prevalence of psychological distress after multivariable adjustment, whereas working hours and caregiving duration were not significantly associated. These findings suggest that restorative free time may warrant further investigation as a potential target for future intervention research. However, because of the cross-sectional design, the temporal and causal direction of these associations cannot be established. Longitudinal and intervention studies are needed to examine these temporal relationships and determine whether changes in restorative free time are associated with subsequent changes in psychological distress.

## Data Availability

The raw data supporting the conclusions of this article are not publicly available because of ethical restrictions and the need to preserve participant anonymity but will be made available by the authors upon reasonable request, without undue reservation.
