# Supplementary material for: Restorative free time and psychological distress among employed family caregivers of individuals with cognitive impairment: a cross-sectional study
Source: Front Psychol. 2026 Oct 26;17:1898843. doi: 10.3389/fpsyg.2026.1898843 (PMC13745454; doi:10.3389/fpsyg.2026.1898843)
Supplement: Supplementary file 1 [file Supplementary_file_1.pdf]

Supplementary Table S1. Sensitivity analyses of the association between daily time use and psychological distress (K6 ≥ 5) without mutual adjustment for other time-use variables

	Age- and sex-adjusted model			Parsimonious model			Fully adjusted model			Patient-factor model			Caregiver-factor model		
**Variable**	**PR**	**95% CI**	**p value**	**PR**	**95% CI**	**p value**	**PR**	**95% CI**	**p value**	**PR**	**95% CI**	**p value**	**PR**	**95% CI**	**p value**
**Restorative free time**															
≥3 h/day	Reference	—	—	Reference	—	—	Reference	—	—	Reference	—	—	Reference	—	—
1–<3 h/day	1.12	0.75–1.69	0.573	1.12	0.75–1.66	0.579	1.08	0.71–1.64	0.720	1.01	0.69–1.49	0.957	1.20	0.79–1.84	0.393
<1 h/day	1.91	1.22–3.01	0.005	1.81	1.11–2.94	0.016	2.16	1.26–3.67	0.005	1.61	0.95–2.74	0.076	2.48	1.49–4.12	<0.001
**Sleep duration**															
<6 h/day	1.80	1.30–2.50	<0.001	1.67	1.20–2.33	0.002	1.85	1.32–2.58	<0.001	1.63	1.18–2.26	0.003	1.96	1.40–2.72	<0.001
6–<8 h/day	Reference	—	—	Reference	—	—	Reference	—	—	Reference	—	—	Reference	—	—
≥8 h/day	1.26	0.72–2.21	0.410	1.18	0.70–1.99	0.530	1.38	0.61–3.16	0.438	1.55	0.59–4.02	0.373	1.18	0.50–2.80	0.709
**Working hours**															
≤6 h/day	Reference	—	—	Reference	—	—	Reference	—	—	Reference	—	—	Reference	—	—
>6–≤8 h/day	0.90	0.61–1.32	0.608	0.88	0.61–1.28	0.501	1.02	0.67–1.56	0.921	0.82	0.57–1.17	0.270	1.06	0.69–1.61	0.805
>8 h/day	1.16	0.70–1.93	0.559	1.20	0.73–1.97	0.467	1.27	0.71–2.28	0.427	0.97	0.60–1.56	0.896	1.31	0.74–2.32	0.352
**Caregiving duration**															
≤1.5 h/day	Reference	—	—	Reference	—	—	Reference	—	—	Reference	—	—	Reference	—	—
>1.5–≤3 h/day	0.82	0.43–1.56	0.543	0.83	0.43–1.59	0.570	0.92	0.54–1.57	0.769	1.01	0.59–1.73	0.975	0.87	0.50–1.51	0.620
>3–≤8 h/day	0.95	0.58–1.54	0.831	0.98	0.60–1.60	0.931	1.07	0.62–1.84	0.819	1.15	0.69–1.91	0.587	1.07	0.62–1.85	0.800
>8 h/day	1.57	0.97–2.58	0.068	1.49	0.83–2.69	0.181	1.63	0.88–3.02	0.118	1.66	0.95–2.90	0.075	1.60	0.97–2.63	0.065

Note: The age- and sex-adjusted model was adjusted for caregiver age and sex. The parsimonious model was additionally adjusted for care recipient diagnosis, MMSE score, level of long-term care certification, and caregiver cohabitation status. The fully adjusted model included care recipient age and sex, caregiver age and sex, care recipient diagnosis, MMSE and PSMS scores, level of long-term care certification, caregiver cohabitation status, marital status, educational level, job type, number of working days, night or shift work, smoking, and alcohol consumption. The patient-factor model included age and sex and care recipient

diagnosis, MMSE and PSMS scores, level of long-term care certification, and caregiver cohabitation status. The caregiver-factor model included age and sex and caregiver marital status, educational level, job type, number of working days, night or shift work, smoking, and alcohol consumption.

None of these models included the other three time-use variables as covariates.

Abbreviations: PR, prevalence ratio; CI, confidence interval; MMSE, Mini-Mental State Examination; PSMS, Physical Self-Maintenance Scale

Supplementary Table S2. Univariable logistic regression analysis of factors associated with restorative free time <1 hour/day

Characteristic	Odds ratio	95% CI	p-value
**Caregiver characteristics**			
Age	1.02	0.96–1.08	0.558
Sex, man	0.42	0.12–1.52	0.187
Currently married	0.42	0.15–1.17	0.098
**Educational level**			
Junior high school/high school	0.376	0.10–1.36	0.135
Vocational school/junior college/technical college	1.63	0.60–4.43	0.334
University/graduate school	1.37	0.48–3.88	0.555
**Job type**			
Mainly desk work	0.56	0.19–1.66	0.299
Mainly talking to people	0.43	0.09–1.93	0.269
Mainly labour	1.97	0.72–5.36	0.184
Number of working days	0.95	0.56–1.60	0.845
Night or shift work	2.74	0.70–10.68	0.146
K6 score	1.12	1.03–1.23	0.010
Cohabitation with care recipient	0.6	0.203–1.77	0.357
Alcohol consumption	0.41	0.15–1.12	0.081
**Care recipient characteristics**			
Age	1.02	0.95–1.10	0.533
Sex, man	0.43	0.12–1.56	0.199
**Diagnosis**			
Alzheimer's dementia	1.41	0.51–3.88	0.505
Mild cognitive impairment	0.68	0.23–2.02	0.493
**Long-term care certification**			
None	0.36	0.11–1.14	0.083
Support level 1–2	0.54	0.12–2.48	0.431
Care level 1–5	3.53	1.25–9.96	0.017
MMSE score	0.97	0.89–1.05	0.420
PSMS score	0.71	0.56–0.90	0.004

Note: Higher PSMS scores indicate better functional status (greater independence in basic activities of daily living). Abbreviations: MMSE, Mini-Mental State Examination; PSMS, Physical Self-Maintenance Scale.

Supplementary Table S3. Spearman rank correlation coefficients among time-use variable

	**Restorative free time**	**Sleep duration**	**Working hours**	**Caregiving duration**
Restorative free time	—			
Sleep duration	0.2506	—		
Working hours	−0.1545	−0.0880	—	
Caregiving duration	0.1188	−0.0760	−0.1534	—

Supplementary Table S4. Characteristics of caregivers included in and excluded from the complete-case analysis

Characteristic	Included (n = 210)	Excluded due to missing data (n = 50)	Missing (n)
Age, mean (SD)	54.3 (8.5)	57.4 (8.1)	0
Sex, men, n (%)	68 (32%)	15 (30%)	0
Currently married, n (%)	159 (76%)	31 (66%)	3
Educational background, n (%)			1
Junior high school/high school	73 (35%)	20 (41%)	
Vocational school/junior college/technical college	76 (36%)	12 (24%)	
University/graduate school	61 (29%)	17 (35%)	
Job type, n (%)			6
Mainly desk work (clerical or computer work)	87 (41%)	9 (20%)	
Mainly talking to people (customer service, sales, selling, etc.)	48 (23%)	19 (43%)	
Mainly labour (work at production sites, physical work, nursing care, etc.)	68 (32%)	14 (32%)	
Other	7 (3%)	2 (5%)	
Number of working days, mean (SD)	4.9 (0.9)	4.8 (1.3)	4
Night or rotating shifts, n (%)	17 (8%)	3 (7%)	5
Psychological distress (K6 ≥5), n (%)	85 (40%)	22 (47%)	3
Living with care recipient, n (%)	84 (40%)	16 (32%)	0
Restorative free time, n (%)			10
<1 h/day	17 (8%)	0 (0%)	
1–<3 h/day	135 (64%)	27 (68%)	
≥3 h/day	58 (28%)	13 (33%)	
Sleep duration, n (%)			3
<6 h	53 (25%)	10 (21%)	
6–<8 h	135 (64%)	30 (64%)	
≥8 h	22 (10%)	7 (15%)	
Working hours, n (%)			6
≤6 h	59 (28%)	11 (25%)	
>6–≤8 h	114 (54%)	24 (55%)	
>8 h	37 (18%)	9 (20%)	
Caregiving duration, n (%)			22
≤1.5 h	52 (25%)	6 (21%)	
>1.5–≤3 h	53 (25%)	9 (32%)	
>3–≤8 h	57 (27%)	7 (25%)	
>8 h	48 (23%)	6 (21%)	
Current smoker, n (%)	28 (13%)	10 (20%)	0
Alcohol consumption, n (%)			1
None	81 (39%)	22 (45%)	
≥1/month	79 (38%)	17 (35%)	
Daily	50 (24%)	10 (20%)	

Note: Percentages for excluded participants are calculated among those with available data for each variable.

Abbreviation: K6, Kessler 6-item Psychological Distress Scale.

Supplementary Table S5. Characteristics of care recipients included in and excluded from the complete-case analysis

Characteristic	Included (n = 210)	Excluded due to missing data (n = 50)	Missing (n)
Age, years, mean (SD)	79.9 (7.3)	80.2 (7.1)	0
Sex, men, n (%)	67 (32%)	10 (20%)	0
Diagnosis, n (%)			1
Alzheimer's dementia	71 (34%)	24 (49%)	
Mild cognitive impairment	78 (37%)	15 (31%)	
Other	61 (29%)	10 (20%)	
Long-term care certification, n (%)			1
None	93 (44%)	21 (43%)	
Support level 1–2	40 (19%)	7 (14%)	
Care level 1–5	77 (37%)	21 (43%)	
MMSE, mean (SD)	21.5 (5.5)	22.1 (4.0)	3
PSMS, mean (SD)	4.5 (1.8)	4.7 (1.9)	6

Note: Percentages for excluded participants are calculated among those with available data for each variable. Higher PSMS scores indicate better functional status (greater independence in basic activities of daily living).

Abbreviations: MMSE, Mini-Mental State Examination; PSMS, Physical Self-Maintenance Scale.
